# Primary Mitral Valve Regurgitation Outcome in Patients With Severe Aortic Stenosis 1 Year After Transcatheter Aortic Valve Implantation: Echocardiographic Evaluation

**DOI:** 10.5935/abc.20170094

**Published:** 2017-08

**Authors:** Thiago Marinho Florentino, David Le Bihan, Alexandre Antonio Cunha Abizaid, Alexandre Vianna Cedro, Amably Pessoa Corrêa, Alexandre Roginski Mendes dos Santos, Alexandre Costa Souza, Tiago Costa Bignoto, José Eduardo Moraes Rego Sousa, Amanda Guerra de Moraes Rego Sousa

**Affiliations:** Instituto Dante Pazzanese de Cardiologia, São Paulo, SP - Brazil

**Keywords:** Mitral Valve Insufficiency, Aortic Valve Stenosis, Transcatheter Aortic Valve Replacement, Echocardiography

## Abstract

**Background:**

Mitral valve regurgitation (MR), present in up to 74% of the patients with severe aortic stenosis (AS), can be a negative prognostic factor when moderate or severe. The outcome of MR after percutaneous transcatheter aortic valve implantation (TAVI) and predictors associated with that outcome have not been well established in the literature.

**Objective:**

To assess the outcome of primary MR in patients submitted to TAVI and to identify associated factors.

**Methods:**

Observational study of patients with symptomatic severe AS submitted to TAVI from January 2009 to April 2015 at two specialized centers. Echocardiographic outcome was assessed with data collected before and 1 year after TAVI.

**Results:**

Of the 91 patients with MR submitted to TAVI and followed up for at least 12 months, 67 (73.6%) had minimum/mild MR before the procedure and 24 (26.4%) had moderate/severe MR. Of those with minimum/mild MR, 62 (92.5%) had no change in the MR grade (p < 0.001), while 5 (7.5%) showed worsening. Of those with moderate/severe MR, 8 (33.3%) maintained the same grade and 16 (66.7%) improved it (p = 0.076). Patients with moderate/severe MR who improved MR grade had lower EuroSCORE II (p = 0.023) and STS morbidity (p = 0.027) scores, as compared to those who maintained the MR grade.

**Conclusion:**

MR grades change after TAVI. This study suggests a trend towards improvement in moderate/severe MR after TAVI, which was associated with lower preoperative risk scores.

## Introduction

Aortic stenosis (AS) is one of the most prevalent heart valve diseases worldwide, being increasingly frequent because of the population ageing.^1^ Data from the American Heart Association have shown a prevalence of AS of 0.4% in the North American population, and of moderate or severe AS of 2.8% in patients older than 75 years.^2^

A new therapeutic option for those patients appeared in 2002, when Cribier et al. performed the first percutaneous transcatheter aortic valve implantation (TAVI).^3^ TAVI has been established as a safe, effective and less-invasive treatment for patients with severe AS and high surgical risk, who used to have no therapeutic alternative for a highly lethal disease.^4^

Mitral valve regurgitation (MR) is commonly associated with AS, whose prevalence can reach up to 74% in the MR population. Literature has shown that approximately 15% of the patients submitted to TAVI have significant MR. The presence of moderate or significant MR can have important implications in deciding between percutaneous or surgical treatment.^5^ While in some studies MR has proved to be an important negative prognostic factor, it has shown no interference with mortality in patients submitted to TAVI in others.^2,6-8^ In most reference centers, significant MR (> 3+) can be a contraindication to TAVI.^9^

Retrospective studies with a limited number of patients have suggested a reduction in MR after TAVI, with better prognosis in patients with smaller residual MR.^10,11^ Some factors have been associated with that improvement, such as low ejection fraction, pulmonary artery pressure under 60 mmHg, and secondary etiology of MR (no structural lesion of the leaflets).^12-15^ However, data on the impact of TAVI in patients with AS and MR in the Brazilian population still lack. In addition, there is no study including only patients with MR of primary etiology.

This study aimed at assessing patients submitted to TAVI who had primary MR associated with AS. We analyzed the clinical and echocardiographic findings of those patients 1 year after TAVI to identify possible factors associated with MR improvement or worsening.

## Methods

This is an observational study including all patients with severe symptomatic AS submitted to TAVI from January 2009 to April 2015 at two centers, where the same multidisciplinary team works, in the city of São Paulo - SP, Brazil. The study project was approved by the Ethics Committee of both institutions. All patients provided written informed consent prior to the TAVI procedure.

Clinical data, such as age, sex, functional class (NYHA) and associated comorbidities, were obtained via complete clinical exam, and the following complementary tests were performed: resting electrocardiography, chest X-ray, laboratory tests, transthoracic echocardiography with protocol to measure the aortic complex, computed tomography angiography of the heart and total aorta, and coronary angiography. On a second assessment, a team of cardiologists specialized in several areas decided which procedure should be performed, its access route and most suitable prosthesis. Intra-operative transesophageal echocardiogram was performed routinely.

In the population studied, the presence of primary MR prior to the transcatheter implantation of the aortic prosthesis and its outcome 1 year after that procedure were assessed. In a secondary analysis, that outcome was correlated with other variables considered to be of clinical importance. Primary MR was defined as that resulting from changes in the tissue constituting any of the mitral valve elements, such as leaflets, ring and subvalvular apparatus, corresponding to valvular calcification, valvular prolapse or rheumatic disease. Secondary MR was defined as that related to left ventricular systolic dysfunction, with no impairment of the valvular tissue itself.

We obtained data from 250 patients classified based on the MR grade. To define the MR severity, effective regurgitant orifice (ERO) and regurgitant volume were determined by using the proximal isovelocity surface area method (PISA), according to the latest American Society of Echocardiography recommendations.^16^

To calculate the ERO and regurgitant volume, the baseline of color flow mapping was lowered to values between 30 and 40 cm/s. The velocity-time integral of the regurgitant jet and the peak velocity of the regurgitant jet were obtained with continuous-wave Doppler, which was also used to measure the mitral transvalvular gradients. The linear measures of the cardiac chambers were obtained in the left parasternal acoustic window (long-axis view), using two-dimensional echocardiography. Left ventricular systolic function was assessed by use of the ventricular volume measures, obtained from the images of the orthogonal apical planes, in an acoustic window from four- and two-chamber view (Simpson’s method). The mitral valve area was calculated by measuring pressure half time (PHT) or with the continuity equation, depending on the case. Pulmonary artery pressure was measured based on the gradient between the right ventricle and the right atrium, obtained with continuous Doppler, and that difference was added to the estimate of the right atrial pressure, determined from the diameter and collapse of the inferior vena cava.

The patients were divided according to the MR severity before and after TAVI into two large groups: trace/mild MR, composed of patients with ERO < 0.2 cm^2^ and regurgitant volume < 30 mL/beat; and moderate/severe MR, composed of patients with ERO > 0.2 cm^2^ and regurgitant volume > 30 mL/beat. Of those groups, we selected 91 patients with primary MR on the pre-procedure echocardiogram who completed 1-year follow-up for analysis of clinical and echocardiographic data.

In all cases, the following characteristics of the procedure were registered: access route; bioprosthesis type and size; and angiographic and echocardiographic results. The patients on vasoactive drugs and/or with hemodynamic instability signs were considered as being critically ill. All patients were cared for by the same medical team, the Heart Team of both hospital centers.

The patients were further divided into four subgroups according to the MR grade before and after TAVI: group 1, patients with moderate/severe MR, who maintained the MR grade after TAVI; group 2, patients with moderate/severe MR prior to the procedure, who changed to trace/mild MR; group 3, patients with trace/mild MR, who remained with the same MR grade after TAVI; group 4, patients with trace/mild MR prior to the procedure, whose MR worsened after the procedure.

### Statistical analysis

Data were recorded in appropriate forms developed for this study, stored in electronic sheets and submitted to statistical analysis. The continuous variables were presented as median and difference between the 25th and 75th percentiles. The categorical variables were presented as absolute numbers and percentages. The continuous variables were compared using the Mann-Whitney test for independent samples, while the categorical variables, by using Fisher exact test or chi-square test. The McNemar test was used to assess the binary categorical variables and their proportion throughout time. All statistical analyses were performed with the SPSS 19 and R programs, 3.1.2 version. The level of statistical significance adopted was 5%.

## Results

**Figure 1:**
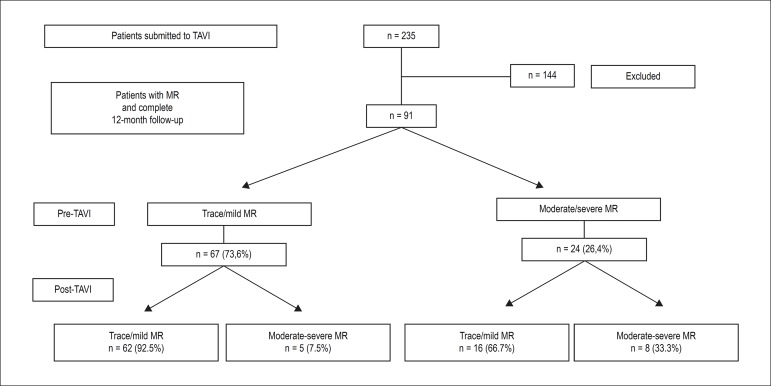
Study design. TAVI: Transcatheter aortic valve implantation; MR: mitral regurgitation.

This study sample comprised 91 patients with MR and submitted to TAVI, who underwent a minimum follow-up of 12 months (Figure 1).

Table 1 shows the demographic data, comorbidities and prognostic scores, and Table 2 shows the echocardiographic parameters of the population studied. Of the 91 patients, 54 (59.3%) were of the female sex, and the median age was 84 (8.25) years. Regarding comorbidities, 33 (36.26%) had significant pulmonary arterial hypertension (systolic pulmonary artery pressure - sPAP > 55 mm Hg), 7 (7.69%) had chronic obstructive pulmonary disease (COPD) and 11 (12.1%) had atrial fibrillation. The medians were as follows: EuroSCORE I, 21.69 (15.39); EuroSCORE II, 5.7 (4.23); STS mortality, 5.65 (4.22); and STS morbidity, 27.25 (11.65); mean aortic gradient, 53 (22.5) mm Hg; left ventricular ejection fraction (LVEF), 62.5 (19)%; left atrial diameter, 45 (9) mm; sPAP, 47.5 (20.75) mmHg; and aortic valve area, 0.7 (0.23) cm^2^. The etiology of primary MR was valvular tissue calcification, including ring and leaflets, in all cases. Mitral stenosis, when present, was mild and related to calcification of the ring and leaflet base, and all patients had a valvular area > 1.5 cm^2^.

**Table 1:** Demographic and clinical data of patients

	Patients (n = 91)	Moderate/severe MR			Trace/mild MR		
		Group 1 (n = 8)	Group 2 (n = 16)	p	Group 3 (n = 62)	Group 4 (n = 5)	p
Age	84 (8.25)	85.5(7.50)	85(10.5)	0.55	84(9.5)	80(21)	0.32
BMI (kg/m^2^)	26.45 (6.01)	23.83(3.87)	26.44(8.10)	0.27	26.67(6.14)	27.73(6.64)	0.82
Female	54(59.3)	3(37.5)	12(75)	0.09	38(61.3)	2(40)	0.64
**Cardiovascular risk factors, n(%)**							
Hypertension	75(82.4)	5(62.5)	13(81.3)	0.36	53(86.9)	3(60)	0.16
Diabetes	23(25.3)	0	2(12.5)	0.53	20(32.8)	0	0.31
Dyslipidemia	60(65.6)	6(75)	9(56.3)	0.65	41(67.2)	3(60)	1.0
**Cardiovascular conditions, n(%)**							
PVD	17(18.6)	1(12.5)	4(25)	0.63	12(19.3)	1(20)	1.0
Carotid lesion > 50%	13(14.2)	2(25)	0	0.10	10(16.1)	1(20)	1.0
PAH > 55 mm Hg	33(36.2)	3(37.5)	7(43.8)	1.0	19(33.8)	3(60)	0.32
Previous stroke	7(7.6)	0	1(6.3)	1.0	5(8.2)	1(20)	0.38
CAD > 50%	43(47.2)	4(50)	6(37.5)	0.67	29(46.7)	4(80)	0.19
Atrial fibrillation	11(12.1)	2(25)	4(25)	1.0	4(6.5)	1(20)	0.33
NYHA, n(%)				1.0			1.0
NYHA FC I/II	20(22.2)	1(12.5)	2(12.5)		16(25.8)	1(20)	
NYHA FC III/IV	71(78.0)	7 (87.5)	14(87.5)		46 (74.2)	4 (80)	
**Non-cardiac conditions, n(%)**							
COPD	7(7.6)	1(12.5)	1(6.2)	1.0	3(4.8)	2(40)	0.04
CrCl < 50 ml/min	59(64.8)	6(75)	9 (56.2)	0.65	40(64.5)	2(40)	1.0
Critical illness	3 (3.3%)	0	0	-	2(3.23%)	1(20%)	0.21
**Risk scores**							
EuroSCORE I	21.69(15.39)	26.91(26.02)	25.13(18.17)	0.35	19.75(11.96)	32.14(19.48)	0.89
EuroSCORE II	5.7(4.23)	8.95(9.84)	4.91(5.23)	0.02	5.63(4.31)	6.6(4.35)	0.63
STS mortality	5.65(4.22)	6.06(6.79)	4.21(5.49)	0.14	5.7(3.40)	5.36(2.23)	0.56
STS morbidity	27.25(11.65)	33.81(14.67)	22.22(10.41)	0.02	26.4(11.29)	31.45(12.76)	0.50
**Type of aortic prosthesis, n(%)**				0.85			0.07
Accurate	24(26.4)	4(50)	5(31.3)		12(19.4)	3(60)	
CoreValve	35(38.5)	2(25)	4(25)		26(41.9)	2(40)	
Sapien XT	32(35.1)	2(25)	7(43.7)		24(38.7)	0	

Data expressed as median (interquartile interval) or frequency (%); MR: mitral regurgitation; Group 1 - patients whose moderate/severe MR remained after transcatheter aortic valve implantation (TAVI); Group 2 - patients whose moderate/severe MR improved to trace/mild after TAVI; Group 3 - patients whose trace/mild MR remained after TAVI; Group 4 - patients whose trace/mild MR worsened to moderate/severe after TAVI; BMI: body mass index; PVD: peripheral vascular disease; PAH: pulmonary arterial hypertension; CAD: coronary artery disease; CrCl: creatinine clearance; COPD: chronic obstructive pulmonary disease; NYHA: New York Heart Association; FC: functional class; STS: Society of Thoracic Surgeons.

**Table 2:** Echocardiographic data of 91 patients with mitral regurgitation (MR) submitted to transcatheter aortic valve implantation and followed up for 1 year

	Patients (n = 91)	Moderate/severe MR			Trace/mild MR		
		Group 1	Group 2	p	Group 3	Group 4	p
		(n = 8)	(n = 16)		(n = 62)	(n = 5)	
LVEF (%)	62.5(19)	47(35.5)	59.5(14.75)	0.358	64(16.50)	67(43)	0.848
LVEDD (mm)	50(10)	53(8.5)	49.5(9.75)	0.326	50(10)	45(24.5)	0.905
LVESD (mm)	31.5(10.25)	31.5(22.25)	32(11.5)	0.620	32(10.25)	27.5(19.75)	0.45
LA (mm)	45(9)	50(6.25)	46.5(9.5)	0.539	43(8)	48(8.5)	0.135
Maximum AoG (mm Hg)	87(34.75)	76.5(42)	81(45.5)	0.603	89(33.5)	78(28)	0.133
Mean AoG (mm Hg)	53(22.5)	46.5(30.75)	49(29.75)	0.520	56(21)	50(18.5)	0.115
AoVA (cm^2^)	0.7(0.23)	0.7(0.33)	0.65(0.30)	0.458	0.7(0.2)	0.7(0.25)	0.578
SPAP (mm Hg)	47.5(20.75)	49(29)	59.5(20.75)	0.391	45(16)	56(14)	0.130
Mitral stenosis (%)	9 (9.9%)	1 (12.5%)	2 (12.5%)	1.0	4 (9.3%)	2 (40%)	0.063
Aortic regurgitation (%)				1.0			1.0
Trace/mild	83 (91%)	7 (87.5%)	15 (93.75%)		56 (90.3%)	5 (100%)	
Moderate/severe	8 (9%)	1 (12.5%)	1 (6.25%)		6(9.7%)	0	
Tricuspid regurgitation (%)				0.829			0.269
Trace/mild	76 (83.5%)	5 (62.5%)	12 (75%)		55 (88.7%)	4 (80%)	
Moderate/severe	12 (13.1%)	3 (37.5%)	4 (25%)		4 (6.5%)	1 (20%)	
Not available	3 (3.4%)	-	-		3 (4.8%)	-	

Data expressed as median (interquartile interval) or frequency (%); LVEF: left ventricular ejection fraction; LVEDD: left ventricular end-diastolic diameter; LVESD: left ventricular end-systolic diameter; LA: left atrium; AoG: aortic gradient; AoVA: aortic valve area; SPAP: systolic pulmonary artery pressure.

The access routes used were as follows: femoral, 77 patients (84%); transaortic, 7 patients; apical, 6; and iliac, 1. The prosthesis types used were as follows: CoreValve, 38.5% of the patients; Sapien XT, 35.1%; and Accurate, 26.4%.

During the 1-year follow-up, 99.9% of the patients were in functional class (FC) I or II, and only one patient was in FC III.

**Figure 2:**
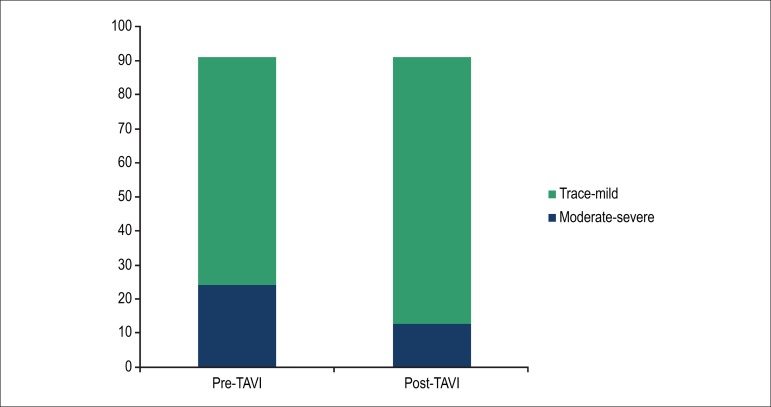
Change in mitral regurgitation grade. TAVI - transcatheter aortic valve implantation.

Of the 91 patients, 67 (73.6%) had trace/mild MR before the procedure, and 24 (26.4%) had moderate/severe MR. Considering the entire group of patients, there was a significant change in the MR grade after TAVI (p = 0.013) (Figure 2).

Of the patients with moderate/severe MR, 8 (33.3%) maintained the same grade and 16 (66.7%) improved their MR (p = 0.076) as shown in Table 3.

**Table 3:** Change in mitral regurgitation (MR) grade in the minimum/mild MR and moderate/severe MR groups

Pre-TAVI	Post-TAVI	N	%	p
**Trace/mild (n = 67)**	Trace/mild	62	92,5	< 0.001
	Moderate/severe	5	7,5	
**Moderate/severe (n = 24)**	Trace/mild	16	66,7	0.076
	Moderate/severe	8	33,3	

TAVI: transcatheter aortic valve implantation.

The patients with moderate/severe MR showed an association between surgical risk, based on the scores, and MR improvement after TAVI. The subgroup with persistent moderate/severe MR had the following medians: EuroSCORE I, 26.91 (26.02); EuroSCORE II, 8.95 (9.84); STS morbidity, 33.81 (14.67); and STS mortality, 6.06 (6.79). The subgroup that improved MR had the following medians: EuroSCORE I, 25.13 (18.17); EuroSCORE II, 4.9 (5.23); STS morbidity, 4.21 (5.49); and STS mortality, 22.22 (10.41). The p values for that difference were 0.35, 0.023, 0.027 and 0.14, respectively.

Regarding the patients with moderate/severe MR, variations in the following echocardiographic parameters were assessed: LVEF; left ventricular end-systolic diameter (LVESD); left ventricular end-diastolic diameter (LVEDD); and left atrial diameter. In group 1, a 0.5-mm reduction in the left atrial diameter was observed on the echocardiogram 1 year after the procedure, and, in group 2, a 4-mm reduction in the left atrial diameter was observed, with statistical significance (p = 0.023 - Table 4).

**Table 4:** Echocardiographic parameters pre-procedure and after 1 year

	Moderate/severe MR			Trace/mild MR		
	Group 1	Group 2	p	Group 3	Group 4	p
LVEF (%)	7(27.75)	3.5(12)	0.51	–0.5(10)	–5(16.5)	0.09
LVEDD (mm)	–1.5(4.25)	0.5(8.75)	0.83	–2(6)	2(11)	0.40
LVESD (mm)	–0.5(3.25)	–1(8.75)	0.31	–0.5(6.63)	6(4.5)	0.06
LA (mm)	–0.5(10)	–4(7.5)	0.02	–1(7)	–1(2.5)	0.54

Data expressed as median (interquartile interval); MR: mitral regurgitation; Group 1 - patients whose moderate/severe MR remained after transcatheter aortic valve implantation (TAVI); Group 2 - patients whose moderate/severe MR improved to trace/mild after TAVI; Group 3 - patients whose trace/mild MR remained after TAVI; Group 4 - patients whose trace/mild MR worsened to moderate/severe after TAVI; LVEF: left ventricular ejection fraction; LVEDD: left ventricular end-diastolic diameter; LVESD: left ventricular end-systolic diameter; LA: left atrium.

Of the patients with -/mild MR before the procedure (n = 67), 92.5% maintained the same MR classification. Worsening to moderate/severe MR was observed in 7.5% of the patients, with a p < 0.01 for remaining in the group with -/mild MR.

Regarding the clinical parameters, in group 3, 4.8% of the patients had COPD. In group 4, 40% of the patients had COPD (p = 0.042), that being the only clinical variable associated with MR change in those patients.

Analyzing the variation in echocardiographic parameters after 1 year, patients with trace/mild MR showed no significant variation between the subgroups (Table 4).

## Discussion

Although some studies have assessed the behavior of MR in patients submitted to TAVI, their results are controversial in establishing if MR improves after aortic prosthesis implantation.^2,6-8^ In addition, no study on that subject has assessed a Brazilian population. A retrospective study analyzing 101 patients with AS undergoing TAVI or surgical valve replacement has reported an improvement in MR grade regardless of the MR etiology.^17^

The present study assessed the behavior of MR in 91 patients submitted to TAVI, who underwent a minimum 12-month follow-up at two large Brazilian centers with the same multidisciplinary team involved in the percutaneous treatment of patients with AS.

The population studied had a median age of 84 years, relatively preserved LVEF, similarly to those of major international studies.^18^ The presence of associated comorbidities, such as systemic arterial hypertension, diabetes *mellitus*, dyslipidemia, peripheral vascular disease, significant carotid disease, coronary artery disease, chronic kidney disease, pulmonary arterial hypertension, COPD and stroke, was similar to that of the major studies.^17^ The median STS score was 5.65. The mean aortic transvalvular gradient was 54.38 ± 16.55 mm Hg (median, 53 mm Hg), higher than that reported in the major studies (mean aortic gradient of 40-50 mm Hg).^17^

The surgical risk assessment of the subgroups was performed by using EuroSCORE I and II, STS mortality and morbidity. In the PARTNER study, the mean STS was 11.8 ± 3.3 in the A cohort and 11.2 ± 5.8 in the B cohort,^19,20^ values relatively higher than the ones observed in this study. Likewise, we observed that the patients selected had lower values in EuroSCORE II. However, it is worth noting that those risk scores are not specific for heart valve disease, and they do not contemplate several comorbidities that influence directly or indirectly the surgical outcome. Thus, those patients can be at high surgical risk and/or have technical difficulties for the traditional approach via sternotomy, even having a low score.^6^

In this study population, 67 (73.6%) patients had -/mild MR before the procedure, while 24 (26.4%) had moderate/severe MR. After valve replacement, 78 (85.7%) patients had minimum/mild MR and 13 (14.3%) had moderate/severe MR. The change in MR severity after TAVI was statistically significant (p = 0.013), showing the impact of aortic valve replacement on MR severity, with a general trend towards MR improvement.

When assessing the subgroup of 24 patients with moderate/severe MR before the procedure, 16 (66.7%) improved MR severity to minimum/mild after TAVI. Although lacking statistical significance, MR improvement can be identified in that specific group. Prospective studies with larger samples are necessary to confirm this trend.

In addition, MR improvement was accompanied by a significant reduction in the left atrial diameter, which can be explained by the reduction of intracavitary filling pressures and regurgitant volume into the atrium. In the general population, the left atrial size is associated with mortality, heart failure and stroke.^21-24^ New studies are required to determine if the left atrial reduction in patients undergoing TAVI, either associated or not with MR change, has prognostic value.

An association was observed between patients with moderate/severe MR who maintained the same MR grade after the procedure and higher surgical risk scores. This might result from the fact that individuals with higher scores have a higher number of chronic mitral valve changes. Studies with larger samples are required to confirm risk scores as independent predictors of the persistence of severe MR after TAVI.

The subgroup with trace/mild MR that worsened after the procedure had a higher prevalence of COPD. A previous publication by the same team has reported COPD as an independent cause of mortality in patients submitted to TAVI, and can represent a critically-ill subgroup,^25^ although the reasons for that finding are not clear. Thus, new studies might clarify that association.

In a recent publication, Kiramijyan et al. have compared the progression of secondary versus primary MR in 70 patients submitted to TAVI, 30 of which had primary MR. The population was assessed 1 month and 1 year after the intervention, and similar survival was evidenced in both groups in the short and long term. Similarly to our findings, in that study MR improved in both groups. However, patients with primary MR had a less marked improvement in MR as compared to those with secondary MR (p = 0.0008).^26^ This emphasizes that patients with primary or secondary MR should be assessed in different ways.

The main limitation of this study relates to the fact that it is a retrospective cohort, with a relatively small number of patients. Therefore, a multivariate analysis to determine independent associations that could justify MR improvement could not be performed. However, we believe this is an important Brazilian study on the subject showing that the percutaneous treatment can be an acceptable therapeutic option in patients with AS, even when there is primary MR associated.

## Conclusion

In this group of patients, a significant change in MR grade was observed after TAVI, those with trace/mild MR maintaining it and those with moderate/severe MR showing a trend towards improvement. In patients with moderate/severe MR, MR grade improvement correlated with lower preoperative risk scores. However, the presence of COPD associated with MR worsening in patients with mild MR before the procedure.
